# Synthesis, characterization and catalytic properties of cationic N-heterocyclic carbene silver complexes

**DOI:** 10.3906/kim-2010-26

**Published:** 2021-06-30

**Authors:** Deniz DEMİR ATLI

**Affiliations:** 1 Manisa Celal Bayar University, Faculty of Science and Arts, Chemistry Department, Manisa Turkey

**Keywords:** N-heterocyclic carbene, silver, dinuclear complex, coupling, propargylamine

## Abstract

Three new dibenzimidazolium salts bridged by 2-methylenepropane-1,3-diyl group were synthesized. Their dinuclear N-heterocyclic carbene Ag(I) complexes were prepared by the reactions of these salts with Ag_2_O. The structures of the synthesized compounds were defined by nuclear magnetic resonance (NMR), Fourier-transform infrared spectroscopy (FT-IR), elemental analysis, and LC-MSMS (for complexes) techniques. Stability of the silver complexes was confirmed by ^1^H NMR spectroscopy. Catalytic activities of Ag(I) compounds were tested for three-component coupling reaction of some aldehydes, amines, and phenylacetylene.

## 1. Introduction

N-heterocyclic carbenes (NHCs) and their transition metal complexes have been very popular in organometallic chemistry for many years. NHC metal complexes with powerful metal-carbon bonds have been typically employed as efficient catalysts in various transformations. NHC-Ag(I) complexes are of capital importance among these complexes. Using of these complexes as carbene transfer reactives for the synthesis of transition metal complexes is one of the most used methods [1–7]. There have been numerous reports related to biological and medicinal applications [8–13]. Also, NHC silver complexes may have luminescence properties [14–17], and these are of significance in material science. NHC-Ag(I) complexes exhibit catalytic efficiencies in cycloaddition of CO_2 _to terminal epoxides [18–20], three component coupling reaction of aldehydes, amines and alkynes (A^3^-coupling reaction) [15,21–32], L-lactide polymerization [33,34], hydration of nitriles [35] and hydroboration of alkynes [36]. 

Dibenzimidazolium salts are featured compounds since NHC procured from these salts can generate easily numerous NHC metal complexes with structural variety. There are many studies related bidentate bis(NHC) ligands in which a linking group acts as a bridge between two NHC units. Silver complexes of these type ligands with particularly antibacterial and antitumor activities are prepared by the reaction of the salts with silver oxide in general [37–39]. Silver complexes containing bis(NHC) and halide ligands (terminal or bridging) form monomeric, oligomeric and polymeric neutral complexes in the solid state [40–42]. Cationic silver bis(NHC) complexes have dinuclear [Ag_2_(NHC)_2_]X_2_ (X = PF_6_ or BF_4_) formulation [43,44]. 

Multicomponent reactions (MCRs) allow the attainment of complex molecules starting from more than two simple building blocks in one step. So, they have importance in various aspects in organic synthesis. A^3^-coupling reaction is one of the best examples of MCRs. This method is a benefical approachment to propargylamines, recurring parts in biologically active compounds and intermediates giving more complex N-heterocycles. As A^3^-coupling has high selectivity for the demanding product and the only by-product is water, it is an encouraging way. Transition metal compounds containing copper, silver, gold, iron, cobalt, and zinc have been frequently used to catalyze this reaction [45–49]. 

In this study, synthesis of three novel 2-methylenepropane-1,3-diyl group bridged dibenzimidazolium salts and dinuclear NHC-Ag(I) complexes are reported. As far as is known, the studies about alkenyl bridged bis(NHC) are relatively less than those of alkyl bridged groups. To synthesize stable metal complexes containing chelating carbene ligands, we have prepared these dinuclear silver complexes. We hope that the metal NHC complexes formed by transmetallation by using these silver NHC complexes can be employed as effective catalysts. Besides, findings regarding catalytic tests of the complexes in A^3^-coupling reactions are presented.

## 2. Experimental

### 2.1. General remarks

All experimental operations were performed in air. The chemicals commercially available were used without any purification. For recording ^1^H (400 MHz) and ^13^C (100 MHz) NMR spectra, a Varian VNMRJ spectrometer was employed. Elemental and mass analyses were executed by a LECO-932 CHNS device and a SHIMADZU LC-MSMS-8040 mass spectrometer, respectively. Thermogravimetric analysis was accomplished using EXSTAR TG-DTA 7300 instrument. IR spectra were obtained with Perkin-Elmer FT-IR spectrophotometer in the range of 400–4000 cm^–1^ using KBr. 

### 2.2. Synthesis of dibenzimidazolium salts

#### 2.2.1. Synthesis of L_1_⋅2HBr (2a)

The mixture of **1** (1 mmol) and 3,5-dimethylbenzyl bromide (2 mmol) was stirred in DMF (3 mL) at 80 °C for 24 h. After cooling to the room temperature and addition of Et_2_O (15 mL), the suspension was filtered. The solid was washed by EtOH (2×5 mL) and Et_2_O (2x5 mL) and dried in air. Yield: 91%. IR n_(NCN)_: 1558 cm^–1^.^1^H NMR (CD_3_OD): d = 9.78 (s, 2H, NCHN), 7.93–7.85 (m, 4H, Ar-H), 7.71–7.63 (m, 4H, Ar-H), 7.13 (s, 4H, Ar-H), 7.05 (s, 2H, Ar-H), 5.65 (s, 4H, NCH_2_), 5.45 (s, 4H, NCH_2_), 5.31 (s, 2H, =CH_2_), 2.29 (s, 12H, Me) ppm. ^13^C NMR (CD_3_OD): d = 139.07, 135.75, 132.61, 131.62, 131.47, 130.49, 127.22, 127.16, 125.90, 118.65, 113.81, 113.35, 50.83, 49.00, 19.83 ppm. LC-MSMS: [M-Br]^+ ^at *m/z* 607.20. Anal. Calc. for C_36_H_38_N_4_Br_2_: C, 62.97; H, 5.59; N, 8.16. Found: C, 63.07; H, 5.42; N, 7.77%. 

#### 2.2.2. Synthesis of L2 ⋅2HBr (2b) 

Compound **2b** was obtained by the reaction of **1** with 3,5-dimethoxybenzyl bromide with the same procedure for **2a** except that drying in vacuum. Yield: 77%. IR: n_(NCN)_: 1554 cm^-1^.^1^H NMR (dmso-d_6_): d = 10.11 (s, 2H, NCHN), 8.03-7.95 (m, 4H, Ar-H), 7.67-7.59 (m, 4H, Ar-H), 6.73 (d, 4H, J = 1.4 Hz, Ar-H), 6.47 (s, 2H, Ar-H), 5.68 (s, 4H, NCH_2_), 5.42 (s, 4H, NCH_2_), 5.27 (s, 2H, =CH_2_), 3.70 (s, 12H, OMe) ppm.^ 13^C NMR (dmso-d_6_): d = 161.29, 143.49, 136.50, 136.27, 131.66, 131.48, 127.29, 127.19, 119.26, 114.48, 114.43, 107.22, 100.42, 55.84, 50.47, 49.22 ppm. LC-MSMS: [M-Br-H]^+ ^at *m/z* 669.20. Anal. Calc. for C_36_H_38_N_4_O_4_Br_2_: C, 57.60; H, 5.11; N 7.47. Found: C, 56.85; H, 4.74; N, 7.38%. 

#### 2.2.3. Synthesis of L3 ⋅2HBr (2c) 

Compound **2c** was obtained by the reaction of **1** with 3,5-di-tert-butylbenzyl bromide with the same procedure for **2a** except that washing process. The solid was washed by Et_2_O (4x5 mL) and dried in air. Yield: 97%. IR n_(NCN)_: 1562 cm^-1^.^1^H NMR (dmso-d_6_): d = 10.17 (s, 2H, NCHN), 8.11 (dd, 2H, J_1_ = 8.6 Hz, J_2_ = 4.5 Hz, Ar-H), 7.99–7.91 (m, 2H, Ar-H), 7.71–7.56 (m, 4H, Ar-H),7.41 (s, 4H, Ar-H), 7.35 (s, 2H, Ar-H), 5.75 (s, 4H, NCH_2_), 5.45 (s, 4H, NCH_2_), 5.09 (s, 2H, =CH_2_), 1.22 (s, 36H, Bu^t^) ppm.^ 13^C NMR (dmso-d_6_): d = 151.56, 143.30, 137.14, 133.55, 131.56, 131.51, 127.27, 127.22, 123.29, 122.64, 114.54, 114.38, 51.01, 49.08, 35.06, 31.58 ppm. LC-MSMS: [M-Br]^+ ^at *m/z* 775.40. Anal. Calc. for C_48_H_62_N_4_Br_2_⋅1.5H_2_O: C, 65.36; H, 7.44; N, 6.35. Found: C, 65.63; H, 7.43; N, 7.32%. 

### 2.3. General procedure for synthesis of NHC-Ag(I) complexes 

The mixture of the salt (1 mmol) and Ag_2_O (2 mmol) was stirred in MeOH (20 mL) at room temperature for 24 h in dark. After filtration through celite, NH_4_PF_6 _(2.5 mmol) in MeOH (10 mL) was added to the filtrate and it was stirred at 25 °C for 2 h in dark. Filtration, washing with MeOH (2 × 5 mL) and Et_2_O (2 × 5 mL), and finally recrystallization from MeCN/Et_2_O (1/3) gave the pure product.

#### 2.3.1 [Ag2(L1)2](PF6)2 (3a) 

Yield: 77%. IR n_(NCN)_: 1400 cm^-1^.^1^H NMR (dmso-d_6_): d = 7.62 (t, 8H, J = 9.1 Hz, Ar-H), 7.34 (s, 8H, Ar-H), 6.76 (s, 12H, Ar-H), 5.55 (s, 8H, NCH_2_), 5.36 (s, 8H, NCH_2_), 4.96 (s, 4H, =CH_2_), 2.00 (s, 24H, Me) ppm.^ 13^C NMR (dmso-d_6_): d = 140.40, 138.31, 136.30, 133.79, 133.68, 129.88, 125.14, 124.90, 124.71, 112.92, 52.23, 52.02, 21.12 ppm. LC-MSMS: [M-PF_6_]^+ ^at *m/z* 1409.35. Anal. Calc. for C_72_H_72_N_8_Ag_2_P_2_F_12_: C, 55.60; H, 4.68; N 7.21. Found: C, 56.53; H, 4.67 ; N, 7.24%.

#### 2.3.2 [Ag2(L2)2](PF6)2 (3b) 

Yield: 76%,. IR n_(NCN)_: 1400 cm^–1^.^1^H NMR (dmso-d_6_): d = 7.68–7.59 (m, 8H, Ar-H), 7.35 (q, 8H, J = 7.5 Hz, Ar-H), 6.27 (d, J = 8.1 Hz, 12H, Ar-H), 5.56 (s, 8H, NCH_2_), 5.38 (s, 8H, NCH_2_), 4.91 (s, 4H, =CH_2_), 3.52 (s, 24H, OMe) ppm.^ 13^C NMR (dmso-d_6_): d = 162.16, 140.46, 138.53, 133.78, 133.71, 124.90, 124.76, 112.85, 105.65, 99.16, 55.42, 52.15, 51.97 ppm. LC-MSMS: [M-PF_6_]^+ ^at *m/z* 1537.40. Anal. Calc. for C_72_H_72_N_8_O_8_Ag_2_P_2_F_12_: C, 51.37; H, 4.32; N, 6.66. Found: C, 52.14; H, 4.16; N, 6.71%.

#### 2.3.3 [Ag2(L3)2](PF6)2 (3c) 

Yield: 70%. IR n_(NCN)_: 1400 cm^-1^.^1^H NMR (dmso-d_6_): d = 7.86 (d, 4H, J = 8.2 Hz, Ar-H), 7.50-7.40 (m, 8H, Ar-H), 7.36 (t, 4H, J = 7.6 Hz, Ar-H), 7.22 (s, 4H, Ar-H), 7.16 (s, 8H, Ar-H), 5.69 (s, 8H, NCH_2_), 5.34 (s, 8H, NCH_2_), 4.74 (s, 4H, =CH_2_), 1.01 (s, 72H, Bu^t^) ppm.^ 13^C NMR (dmso-d_6_): d = 151.19, 135.72, 133.95, 133.50, 125.06, 124.93, 122.19, 121.92, 113.28, 112.72, 52.75, 34.78, 31.36 ppm. LC-MSMS: [M-PF_6_]^+ ^at *m/z* 1746.60. Anal. Calc. for C_96_H_120_N_8_Ag_2_P_2_F_12_: C, 60.94; H, 6.41; N, 5.92. Found: C, 61.30; H, 6.41; N, 5.69%.

### 2.4. General procedure for A3-coupling reaction

NHC silver complex (3 mol%), aldehyde (1 mmol), amine (1.2 mmol) and phenylacetylene (168 mL, 1.5 mmol) were placed in a test tube with screw cap. The mixture was stirred at 80 °C for 18 h in dark medium. After cooling to room temperature, Et_2_O and MgSO_4_ were added to the mixture. Filtration was done and Et_2_O was removed from the filtrate. Related propargylamine was obtained in pure form by column chromatography. 

## 3. Results and discussion

**Figure 1:**
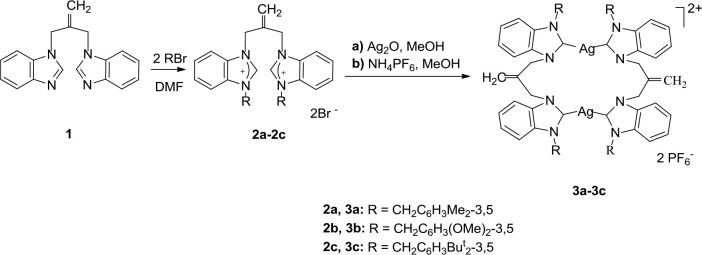
Synthesis of **2a-2c** and **3a-3c**.

Synthesis methods of dibenzimidazolium salts and dinuclear NHC-Ag(I) complexes are in Figure 1. Dibenzimidazole compound **1** was prepared by the reaction of two equivalents of benzimidazole and one equivalent of 1,1-bis(chloromethyl)ethylene by using NaH base in THF [15]. Quaternization of **1** with two equivalents of substituted benzyl bromides afforded the dibenzimidazolium dibromide salts **2a-2c** . Transition metal complexes of N-benzylic benzimidazol-2-ylidene are of importance in organometallic chemistry, and there have been various studies on these compounds [50,51]. Dinuclear cationic NHC silver hexafluorophosphate complexes **3a-3c** were procured by the reactions of **2a-2c** with two equivalents of Ag_2_O and then salt metathesis reactions of bromide complexes with NH_4_PF_6_ in methanol medium. 

The results of elemental analysis confirm the expected formulations. While the signals of the acidic C2 protons of **2a-2c** appear at 9.78-10.17 ppm in the ^1^H NMR spectra, these signals resonating in a low field do not exist in those of the Ag(I) complexes. This observation points out formation of a NHC metal complex as previously reported [52]. The absence of carbene carbon signals in the ^13^C NMR spectra of the silver complexes may be attributed to the fluxional behaviour of the NHC silver complexes [15,53]. IR peaks concerning the stretching vibrations of -C=N- groups for the salts are present at 1554–1562 cm^–1^. Whereas, these values decrease to 1400 cm^–1^ for the metal complexes. These data are compatible with the literature [54]. The stretching frequencies related to P-F bond for the complexes appear in the range of 834–840 cm^–1^. The sharp band observed in 3390 cm^-1^ for **2c** is assigned to the n(O-H) of hydrated water. TGA/DTA analysis supports that this compound is a hydrate molecule. Unfortunately, single crystals required for XRD analysis were not obtained despite all efforts. The molecular weights of **2a-2c** and **3a-3c** were proved by LC-MSMS spectroscopic analysis. [M-Br]^+^ and [M-2Br]^+^ peaks are observed for **2a-2c** . There are [M-PF_6_]^+^ signals at 1409.35, 1537.40, and 1746.60, respectively in the mass spectra of **3a-3c** . Mass data affirm dinuclear [Ag_2_(L)_2_](PF_6_)_2_ formulation. It is believed that the cationic silver complexes **3a-3c** isolated as hexafluorophosphate salts do not form polymers. 

Stabilities of the silver complexes in solution were studied by ^1^H NMR spectroscopy for a period of ten days. ^1^H NMR spectra were recorded on the day their dmso-d_6_ solutions were prepared and after one, four, seven, and ten days. The spectra for stability testing are shown in Figures S7-S9 in supporting information. The results evidently point out that the complexes are stable in solution even after ten days. 

While A^3^-coupling reactions have been catalyzed by many transition metal ions, the number of studies on using NHC silver complexes is limited. In this work, catalytic activities of Ag(I) compounds were studied for three-component coupling reaction of some aldehydes, amines and phenylacetylene. The reaction of p-formaldehyde, diethylamine, and phenylacetylene was carried out using different solvents and different amount of catalyst **3a** (Table 1, entries 1-8). The results showed that solvent free medium and increased amount of catalyst raised the activity. N,N-diethyl-3-phenylprop-2-yn-1-amine was obtained in 78% yield with 3 mol% catalyst (Table 1, entry 3). When the same reaction was performed by using piperidine instead of diethylamine, 51%–52% yields were obtained (Table 1, entries 11-13). These data are comparable with the literature [25,30]. It was understood that each of the three complexes exhibited similar activities in both reactions examined. In our previous work, 59% yield was obtained for this reaction with a similar complex containing 3-methoxybenzyl group on NHC ligand [15]. The presence of a larger number of alkyl groups on the benzyl substituent causes a decrease in the catalytic activity. 3,5-dimethylbenzyl, 3,5-dimethoxybenzyl and 3,5-di-tert-butylbenzyl substituents on NHC ligands did not affect the catalytic behaviours of the catalysts. This consequence is consistent with the literature [23,55]. In the case of using aliphatic aldehydes and the amines, such as diethylamine and piperidine, the propargylamine compounds were gained in moderate yields (Table 1, entries 14,15). Using morpholine caused low yields (Table 1, entries 16–18). When the results are compared, it is seen that the prepared complexes show less activity than monomeric NHC silver complexes possibly because of steric hindrance. This result is consistent with the literature [31].

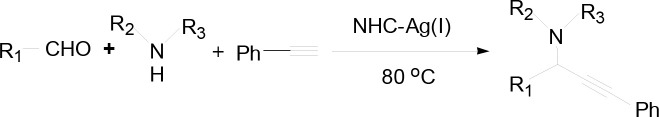



**Table 1:** NHC silver catalyzed A3-coupling reaction a

Entry	Catalyst (% mol)	Solvent	Aldehyde	Amine	Yield (%)b,c
1	3a (1)	-	HCHO	NHEt2	42
2	3a (2)	-	HCHO	NHEt2	64
3	3a (3)	-	HCHO	NHEt2	78
4	3a (3)	water	HCHO	NHEt2	15
5	3a (3)	toluene	HCHO	NHEt2	18
6	3a (3)	DMF	HCHO	NHEt2	24
7	3a (3)	acetone	HCHO	NHEt2	45
8	3a (3)	MeCN	HCHO	NHEt2	62
9	3b (3)	-	HCHO	NHEt2	80
10	3c (3)	-	HCHO	NHEt2	80
11	3a (3)	-	HCHO	piperidine	51
12	3b (3)	-	HCHO	piperidine	51
13	3c (3)	-	HCHO	piperidine	52
14	3a (3)	-	CH3(CH2)4CHO	NHEt2	44
15	3a (3)	-	C6H11CHO	piperidine	68
16	3a (3)	-	HCHO	morpholine	14
17	3a (3)	-	CH3(CH2)4CHO	morpholine	26
18	3a (3)	-	PhCHO	morpholine	10

a Reaction conditions: Aldehyde (1.0 mmol), amine (1.2 mmol), phenylacetylene (1.5 mmol), catalyst, 18 h, 80 °C, in air.b Isolated yields.c Average of two runs.

**Figure 2:**
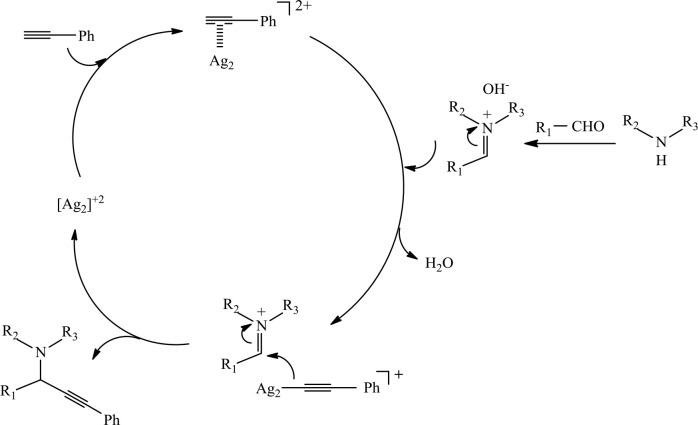
Proposed mechanism of propargylamine formation.

Based on the literature [56–58], a mechanism can be proposed (Figure 2). Firstly, C-H activation of phenylacetylene forms a silver-acetylide complex and acidic proton. The formation of this complex may proceed through a p-complex. Then, in situ formed silver acetylide reacts with iminium cation to give propargylamine and the catalyst.

## 4. Conclusion

A new series of dibenzimidazolium salts bridged by 2-methylenepropane-1,3-diyl group and their dinuclear NHC-Ag(I) complexes were synthesized and characterized. Preliminary catalytic tests for A^3^-coupling reactions of some aldehydes, amines and phenylacetylene were performed. The results deduced that **3a-3c** exhibited similar activities, and the substituents on NHC ligands in the catalysts did not change the yields. Preparation of different transition metal complexes obtained from these compounds and their catalytic experiments have proceeded.
